# Novel Implication of the Basement Membrane for Breast Cancer Outcome and Immune Infiltration

**DOI:** 10.7150/ijbs.81939

**Published:** 2023-03-05

**Authors:** Wenwen Tian, Yongzhou Luo, Yuhui Tang, Yanan Kong, Linyu Wu, Shaoquan Zheng, Yutian Zou, Chao Zhang, Jindong Xie, Xinpei Deng, Junsheng Zhang, Nian Chen, Xiaoming Xie, Weidong Wei

**Affiliations:** 1 State Key Laboratory of Oncology in South China, Sun Yat-Sen University Cancer Center, Collaborative Innovation Center for Cancer Medicine, 651 East Dongfeng Road, Guangzhou, 510060, China.; 2 Breast Disease Center, The First Affiliated Hospital, Sun Yat-sen University, Guangzhou, 510080, People's Republic of China.; 3 The First Affiliated Hospital, Hengyang Medical School, University of South China, Hengyang, 421001, China.

**Keywords:** breast cancer, prognosis, basement membrane, immune infiltration.

## Abstract

Therapeutic failure in breast cancer patients is largely attributed to postoperative advancement and therapy resistance. Nevertheless, an efficacious prognostic signature for recognizing this population is lacking. The basement membrane (BM) has been proven to be strongly involved in cancer progression and metastasis, and has the potential to be a powerful predictor in breast cancer. In this study, substantial bulk RNA transcriptomics, single cell RNA transcriptomics and clinical information were collected from TCGA-BRCA, METABRIC and GSE96058, and Kaplan-Meier survival curves, single cell analysis and *in vitro* experiments were conducted to validate the signature. From the results, a prognostic index, namely, the BMscore, was established with six pivotal BM genes, specifically LOXL1, FBLN1, FBLN5, SDC1, ADAMTS8 and PXDNL. Verification by independent cohorts showed that breast cancer patients with high BMscore had a distinctly worse outcome. By integrating the BMscore and clinical factors, we constructed a prognostic nomogram that displayed good predictive capability. Furthermore, we evaluated the implication of the BMscore in breast cancer immune infiltration. More importantly, a strongly positive correlation between the BMscore and EMT activity was revealed with immunohistochemistry and *in vitro* experiments. Taken together, we provided a novel BMscore gene signature for breast cancer patients to predict clinical prognosis and metastasis accurately, which may help with individualized clinical decision-making.

## Introduction

Occurring with the highest incidence among female worldwide, a portion of breast cancer patients still fail to respond well to standard and multidisciplinary treatment, which leads to a dismal prognosis with recurrence and metastasis 1-3. There is increasing evidence that molecular biomarkers could be applied to identify high-risk subpopulations and predict prognosis in breast cancer patients 4-6. However, there is still a lack of clinically useful biomarkers to facilitate individual treatment for breast cancer. Therefore, more reliable predictive biomarkers are essential for improving patient diagnosis and treatment in breast cancer.

The extracellular matrix (ECM) plays a core role in the tumor microenvironment 7. As a barrier in the ECM, the basement membrane (BM) restricts the distant dissemination of cancer cells 8. As such, abnormal regulation of the BM could promote cancer invasion and metastasis 9. Extensive studies have shown that BM related genes are associated with the prognoses of various tumors, including renal cell carcinoma 10-12 and bladder cancer 13. Regrettably, exploration of BM related genes in breast cancer is still lacking.

Emerging evidence suggests that breast cancer can develop and progress through both tumor cells and a significantly altered microenvironment surrounding them 14. Studies have proposed that the ECM serves as a key player in tumor proliferation and migration due to its dynamics and versatility 15. Altered ECM metabolism in cancer, such as collagen IV degradation leading to tumor invasion via the BM, was usually induced by immune cells and cancer-associated fibroblasts 16. The aforementioned findings implied a potential link between the BM and tumor immune infiltration, which has not yet been elaborated clearly.

Epithelial-mesenchymal transition (EMT) of the BM converts tumor cells from an epithelial phenotype to a mesenchymal-like phenotype and enables lymphovascular metastasis, which ultimately facilitates cell settlement and metastasis in distant organs 17. Multiple BM proteins have been found to be overexpressed in breast cancer and to promote tumor invasion through EMT 18. In this context, it is imperative to illustrate the association between the BM and EMT and to thus, provide more evidence for their critical role in breast cancer.

Given the critical role of the BM in cancer, we constructed a prognostic BM-related index (BMscore) according to BM-related genes. Furthermore, multiple analyses based on the signature were performed, including survival analysis, functional enrichment analysis, estimation of tumor immune infiltration and correlation to EMT.

## Methods

### Data sources of the bioinformatics analyses and tissue collection

Four sources of data that were collected in our study, including The Cancer Genome Atlas (TCGA) database (113 normal and 1,113 breast cancer samples), the Molecular Taxonomy of Breast Cancer International Consortium (METABRIC) database (1,904 breast cancer samples), GSE96058 (3,409 breast cancer samples), and GSE176078 (26 breast cancer samples). Bulk RNA and single-cell RNA transcriptomic profiles and relevant clinical information of all tumor samples were downloaded and subsequently we obtained three independent datasets after filtering with the following exclusion criteria: 1) no survival information or overall survival less than 90 days; and 2) no surgical process. To identify BM related genes, we referred to a recently published report that comprehensively delineated a network of 222 human proteins and their animal orthologs localized to BMs 19. And we selected totally 203 BM matrix protein and cell surface interactor genes confirmed in humans for further analysis. Tumor specimens of breast cancer patients for RT-qPCR and immunohistochemistry (IHC) were retrospectively obtained from the Sun Yat-Sen University Cancer Center (SYSUCC) with informed consent provided by each patient. And also, this study was endorsed by the SYSUCC Research Ethics Committee (No. 2021-358).

### Selection and understanding of candidate BM genes

First, differential expression analysis between normal and breast cancer samples from TCGA-BRCA was performed with the R package “edgR” 20 and displayed by heatmap and volcano plots. Somatic mutations of differentially expressed BM genes were visualized using the R package “maftools” 21. Then, to further screen core BM genes, univariate Cox regression analysis was applied to determine OS-related genes. Finally, using the “glmnet” R package, the least absolute shrinkage and selection operator (LASSO) Cox regression analysis was employed to single out the optimal BM genes 22. To obtain more details of these candidate BM genes, we used the R package “RCircos” to visualize their mRNA expression levels and locations on the chromosomes and the correlations between these genes and other genes were shown with strings 23. Moreover, we explored the correlation features, expression levels and Kaplan-Meier survival curves among the six BM genes.

### Establishment and validation of the BMscore signature

To establish a prognostic index for predicting the overall survival probabilities, the BMscore of each breast cancer patient was obtained by the following formula:

BMscore = 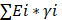
(where Ei represents the mRNA expression level of each BM gene; andγi represents the corresponding regression coefficient). Then, patients in each dataset were classified into the high BMscore and low BMscore groups by the median value. Principal component analysis (PCA) was performed to test the clustering effect of the BMscore, and Kaplan-Meier survival curves were plotted to determine the survival prediction function of the BMscore in all three cohorts in the study.

### GSEA and evaluation of tumor immune infiltration

Gene set enrichment analysis (GSEA) was performed to characterize the biological functions of the high and low BMscore groups, which involved “h.all.v7.5.1.entrez.gmt” [HALLMARK], “c5.all.v7.5.1.entrez.gmt” [GO] and “c2.cp.kegg.v7.5.1.entrez.gmt” [KEGG] as the reference database with |NES| > 1.5 and FDR q-value < 0.1^24^. The evaluation of tumor immune infiltration in different BMscore subgroups was carried out with the ESTIMATE algorithm 25 and the CIBERSORT algorithm 26.

### Nomogram building and assessment based on the BMscore

Univariate and multivariate Cox regression analyses of OS were performed to verify that the BMscore could be regarded as an independent prognostic indicator for breast cancer patients. Next, a clinical nomogram associated with the BMscore was constructed by the R packages “rms” and “regplot” 27, and subsequently assessed by calibration curves, decision curve analysis (DCA) and the receiver operating characteristic (ROC) curves 28-30.

### Cell culture and siRNA transfection

Breast cancer cell lines, including MCF-10A, MDA-MB-231, BT-549, SKBR3, MCF-7 and T47D, were all purchased from ATCC and cultured as indicated by standard procedures 31. And siRNA transfection was performed using Lipofectamine™ 3000 Reagent (Invitrogen, USA) following the product instruction (Ribio, China). The siRNA sequences targeting SDC1were as follows: siRNA#1-GCAAGATATCACCTTGTCA; siRNA#2-GGGAGAATACGGCTGTAGT.

### RNA extraction, cDNA synthesis and RT-qPCR

The extraction of total RNA from frozen breast cancer tissue sections was performed with TRIzol reagent (Invitrogen, USA) while that from breast cancer cells was processed with an RNA quick extraction kit (Qiagen, China). Then, RNA samples were reverse transcribed to cDNA and RT-qPCR was performed using the PrimeScript RT Reagent Kit and the Takara RT-PCR kit (Takara, Japan) according to the manufacturer's instructions. With normalization to β-actin, all target gene expression levels were calculated with the 2^-ΔΔCt^ method after reactions were repeated in triplicate. The primer sequences are as follows: ADAMTS8-F: 5′- ACCAAGCGGTTTGTGTCTGAG-3′and ADAMTS8-R: 5′-AGAAGTTACGCAGTGTAAGCC -3′; FBLN1-F: 5′-GTGGTATTCATAACTGCCTCCC-3′and FBLN1-R: 5′-CTCCTCGTTGAGATGGTAGCC-3′; FBLN5-F: 5′-TCGCCAGTCAGGACAGTGT-3′ and FBLN5-R: 5′-AGTAGGGGTTCGAGTAGGGC-3′; PXDNL-F: 5′-GAGACCTTCTGAGATTAGAGCGA-3′ and PXDNL-R: 5′-GCGTTGGAATCCAGACGCA-3′; LOXL1-F: 5′-CTGTGCTGCGGAGGAGAAG-3′ and LOXL1-R: 5′-GTAGTGGCTGAACTCGTCCA-3′; and SDC1-F: 5′-ACGGCTATTCCCACGTCTC-3′ and SDC1-R: 5′-TCTGGCAGGACTACAGCCTC-3′.

### Immunohistochemistry (IHC) and Western blot (WB)

For IHC of breast cancer tissues, a standard procedure was performed as previously described 32, and we used a semiquantitative grading system (the H-score) to compare immunohistochemical staining intensities 33. In addition, cells were harvested for western blotting to detect the protein expression levels of target genes as described before 34.

### Cell viability and migration assays

The CCK8 assay was applied to assess cell viability while the scratch and transwell migration assays were conducted for cell migration. Typically, these assays were undertaken following previously published protocols 35. Data are represented as the mean±s.d. of three independent experiments for cell migration and proliferation.

### Statistical methods

All statistical analyses in the study were performed using R software (Version 4.2.0) and GraphPad Prism (Version 8.0) software. Comparisons between different groups were carried out using t tests (two groups) or one-way ANOVA (multiple groups). The correlation coefficient was assessed by Spearman's test. Statistical significance set at p < 0.05, ns > 0.05, and denoted by asterisks (*p<0.05, **p<0.01, ***p<0.001 and ****p<0.0001).

## Results

### Selection strategy for prognostic BM genes to establish the BMscore

**Figure 1:**
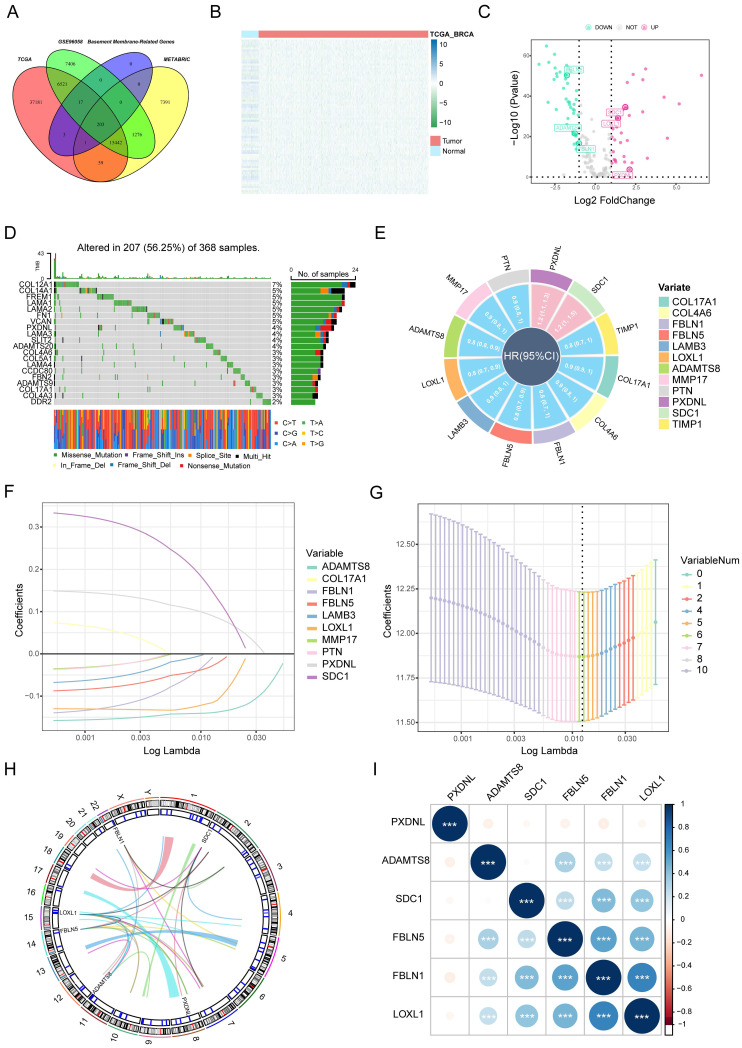
Selection of candidate BM genes in breast cancer. (A) Venn diagram showing the BM genes shared by TCGA-BRCA, METABRIC and GSE96058. (B and C) Heatmap (B) and volcano plot (C) of differentially expressed BM genes identified in TCGA-BRCA. (D) Diagram of somatic mutations for the top 20 BM genes. (E) Plot visualizing the univariate analysis of OS in TCGA-BRCA. (F and G) Six signature BM genes selected by LASSO Cox regression analysis. (H) Circos plot depicting the locations and expression levels of the six signature genes. (I) Correlation plot for the six signature genes in breast cancer.

Through the intersection of the BM gene set and three datasets used in the study, we obtained 203 BM genes **(Figure 1A)**. Then, from dozens of candidates, we carried out differential expression analysis (|log2FC|>1 and FDR<0.05) and obtained 77 BM genes that were obviously upregulated or downregulated in TCGA-BRCA. The results are displayed in the form of a heatmap **(Figure 1B)** and a volcano plot **(Figure 1C)**. Meanwhile, an oncoplot illustrated the top 20 BM genes which had the most somatic mutations **(Figure 1D)**. Then, we developed a univariate analysis for OS in TCGA-BRCA to acquire 12 BM genes that would be more likely to be essential for further consideration **(Figure 1E)**. More importantly, six pivotal BM genes were identified using LASSO Cox regression analysis, including LOXL1, FBLN1, FBLN5, SDC1, ADAMTS8 and PXDNL **(Figure 1F, G)**.

**Figure 2:**
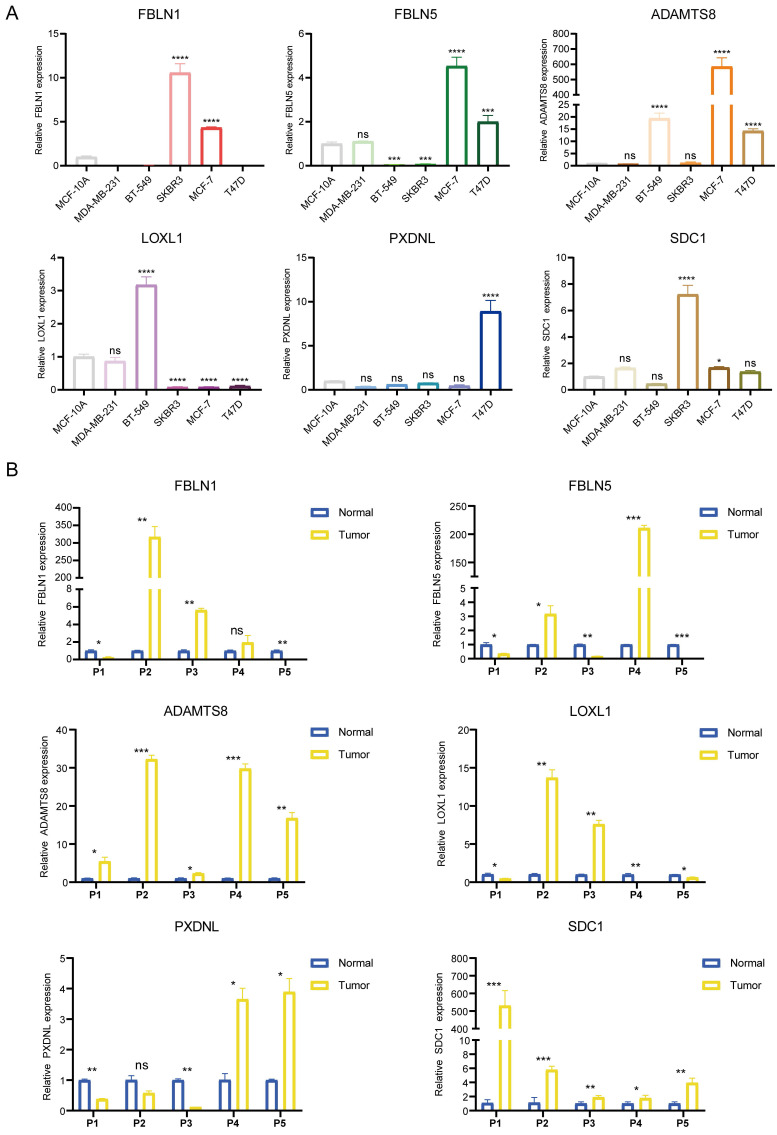
Determination of the mRNA expression levels of the 6 signature genes. (A and B) The relative expression of each gene was quantified with histograms and normalized to β-actin in cell lines (A) and tissues (B) from breast cancer patients.

As the key marker genes for further dissection, we explored their correlations, expression levels and prognoses in TCGA-BRCA individually. First, a Circos plot demonstrated the chromosomal locations and expression levels of 6 BM genes while an interaction network summarized the correlations of the indicated genes **(Figure 1H, I)**. Based on the TCGA-BRCA dataset, we found that LOXL1, SDC1 and PXDNL were highly expressed in breast cancer while the expressions levels of FBLN1, FBLN5 and ADAMTS8 were lower than those in normal breast samples **(Figure S1A)**. The decreased or increased expressions of the 6 BM genes was further validated in breast cancer cell lines and matched breast cancer tissues by RT-qPCR **(Figure 2A, B)**. For each gene, there exhibited divergent expression levels according to different molecular subtypes of breast cancer.

In Kaplan-Meier survival analyses for OS in TCGA-BRCA, the survival of breast cancer patients exhibiting high expression of PXDNL (p<0.0001) and SDC1 (p=0.0007) as well as low expression of FBLN1 (p=0.0025), FBLN5 (p=0.0029), LOXL1 (p=0.00037) and ADAMTS8 (p<0.0001) was significantly more unfavorable **(Figure S1B)**. For disease-free survival (DFS) analysis** (Figure S2A)**, we found that higher levels of FBLN5 (p=0.014), PXDNL (p=0.0065) and SDC1 (p<0.0001), as well as lower levels of FBLN1 (p=0.023), FBLN5 (p=0.014) and ADAMTS8 (p=3e-04) were associated with worse outcomes. Similarly, breast cancer patients with increased PXDNL (p=0.00011) and SDC1 (p<0.0001), and decreased FBLN1 (p=0.033) and ADAMTS8 (p=0.00017) expression levels tended to have shorter disease-specific survival (DSS) time **(Figure S2B)**. Even more, Kaplan-Meier survival curves of progression-free survival (PFS) demonstrated inferior survival probabilities in patients with high tumor expression of PXDNL (p=0.0001) and SDC1 (p<0.0001) and low tumor expression of FBLN1 (p=0.0087), FBLN5 (p=0.026) and ADAMTS8 (p<0.0001) **(Figure S2C)**. In the bulk of survival analyses, it is reasonable to consider PXDNL and SDC1 as critical components of the BMscore signature. Ultimately, a BM-related prognostic index, the BMscore, was generated with the following formula: BMscore = Expression of SDC1 * 0.1625 + Expression of PXDNL * 0.0942 - Expression of ADAMTS8 * 0.1399 - Expression of LOXL1 * 0.1176 - Expression of FBLN5 * 0.0328 - Expression of FBLN1 * 0.0189.

### The effectiveness of the BMscore in terms of predicting the prognoses of breast cancer patients

**Figure 3:**
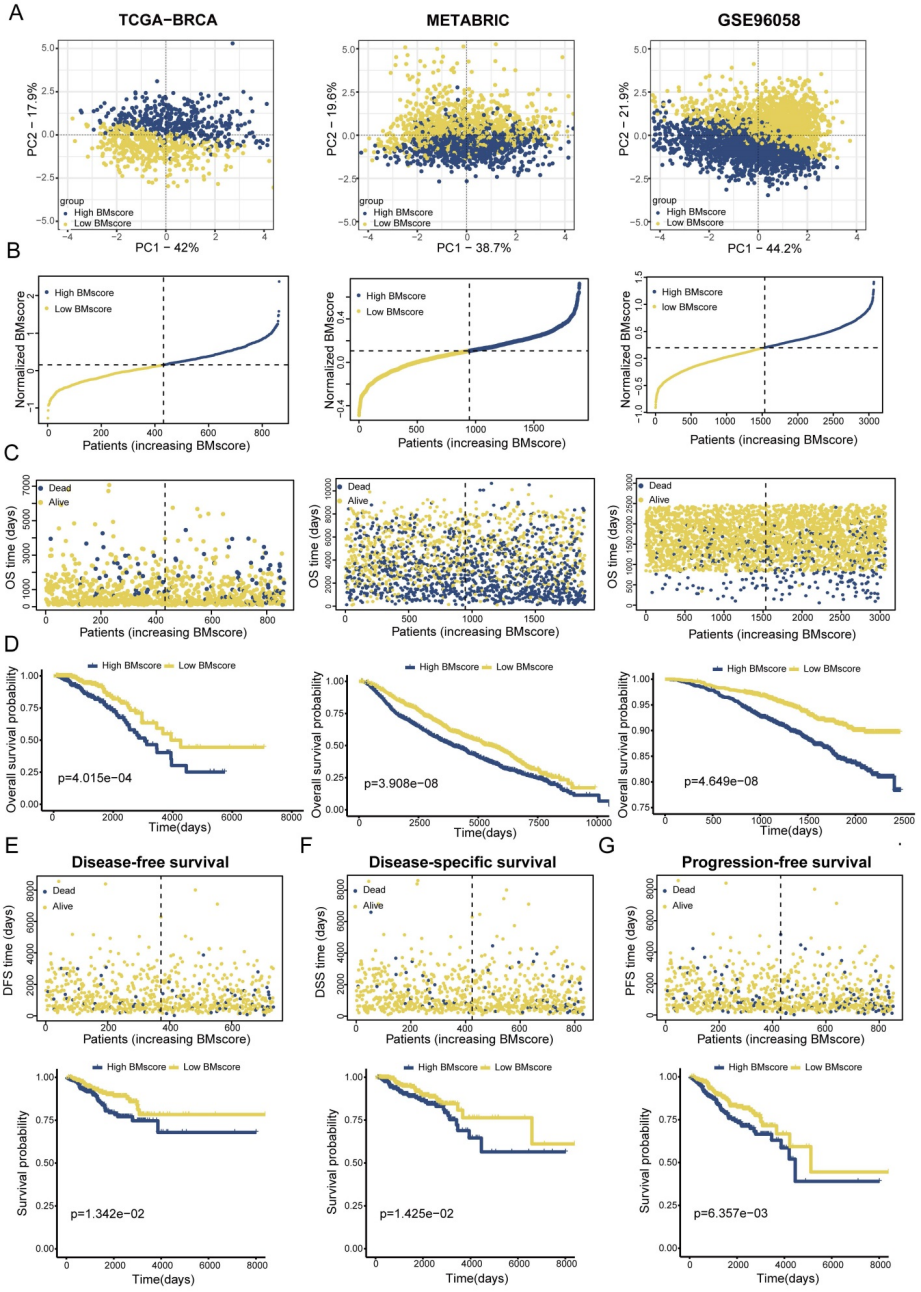
Establishment and validation of a BM-related signature to predict clinical outcome for breast cancer patients. (A) Principal component analysis (PCA) based on BMscore levels in TCGA-BRCA, METABRIC and GSE96058. (B and C) Distribution of patient survival status and survival time according to the BMscore in TCGA-BRCA, METABRIC and GSE96058. (D-G) Overall survival curves in TCGA-BRCA, METABRIC and GSE96058 (D), and KM-plotter curves of disease-free survival (E), disease-specific survival (F) and progression-free survival (G) in TCGA-BRCA.

To validate the applicability of the BMscore in predicting survival probability among breast cancer patients, patients from the TCGA-BRCA training set and two validation sets (METABRIC and GSE96058) could be defined as high and low BMscore subgroups respectively according to the median value and were visualized with two-dimensional PCA plots **(Figure 3A)**. As expected, the deaths of breast cancer patients increased significantly with rising BMscores across all cohorts **(Figure 3B, C)**. Moreover, patients with higher BMscores had worse OS probability in the KM analyses (**Figure 3D**, TCGA-BRCA, p=4.015e-04; METABRIC, p=3.908e-08; GSE96058, p=4.649e-08), which further reinforced the above conclusion. As BM has been reported to be involved in tumor invasion and metastasis, we also performed KM survival analyses of DFS, DSS and PFS in TCGA-BRCA. From the results, significantly increased deaths were observed in breast cancers with high BMscores, which indicated more unfavorable DFS **(Figure 3E**, p=1.342e-02**)**, DSS **(Figure 3F**, p=1.425e-02**)** and PFS **(Figure 3G**, p=6.357e-03**)**.

### Comprehensive insights into the BMscore signature involved in breast cancer

**Figure 4:**
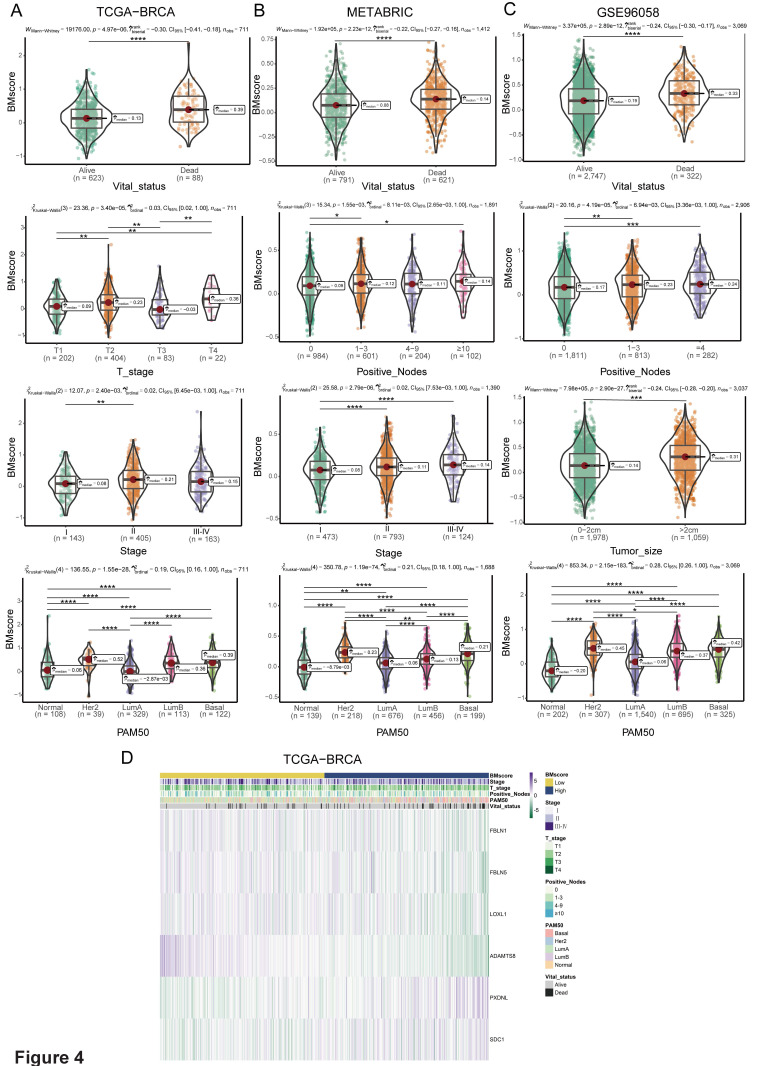
Correlation between the BMscore and clinical indicators in breast cancer. (A, B and C) Boxplots illustrating the alteration of clinical indicators according to the BMscore levels in TCGA-BRCA (A), METABRIC (B) and GSE96058 (C). (D) Heatmap displaying the relationship between the BMscore and clinical factors in TCGA-BRCA.

Since BMscore has been proven to have predictive value for the clinical outcomes of breast cancer patients, we further uncovered the associations of the BMscore with additional features in breast cancer. For clinicopathological parameters, the findings demonstrated that the BMscore had marked correlations with T stage, stage and molecular subtypes in TCGA-BRCA **(Figure 4A, D)**, positive nodes, stage and subtypes in METABRIC **(Figure 4B)**, and positive nodes, tumor size and subtypes in GSE96058 **(Figure 4C)**. Collectively, significant relationships emerged between the BMscore levels and clinical features in breast cancer, which implied that patients with high BMscores tended to be more likely to have lymph node metastases and severe clinical stage.

**Figure 5:**
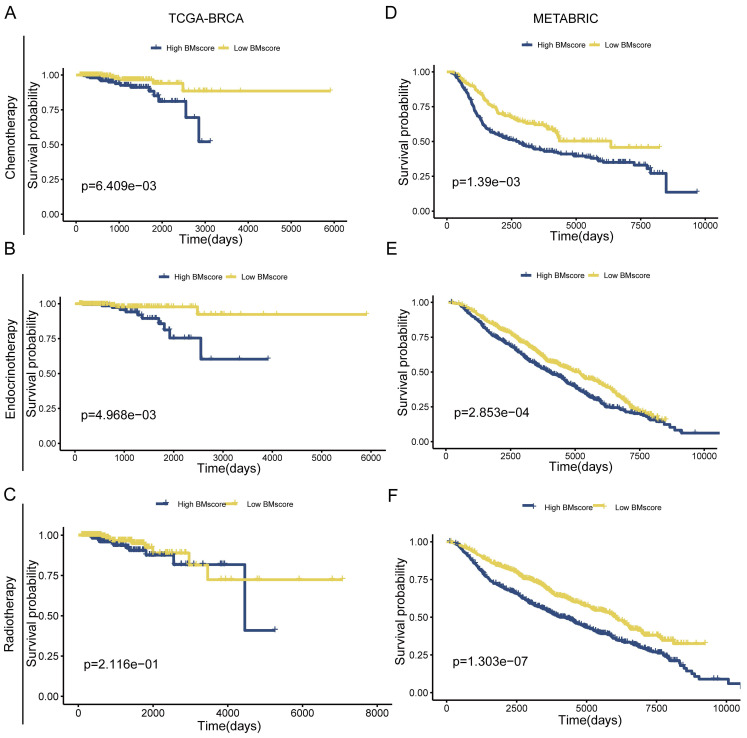
The implication of the BMscore on breast cancer treatment resistance. (A, B and C) The relationship between the BMscore and treatment sensitivity in TCGA-BRCA, including chemotherapy (A), endocrine therapy (B) and radiotherapy (C). (D, E and F) The relationship between the BMscore and treatment sensitivity in METABRIC, including chemotherapy (D), endocrine therapy (E) and radiotherapy (F).

Due to poor response to adjuvant therapy after radical surgery, the survival time of some patients usually tends to be shorter. Hence, we explored whether the BMscore has the capacity to predict the response to the clinical treatment in breast cancer. The result from TCGA-BRCA presented an unexpected finding suggesting that significantly positive correlations were found with a high BMscore and therapy resistance to chemotherapy **(Figure 5A**, p=6.409e-03**)** and endocrinotherapy **(Figure 5B**, p=4.968e-03**)** in addition to radiotherapy **(Figure 5C**, p=2.116e-01**)**. Likewise, the validation results of METABRIC showed that the high BMscore population was much less responsive to chemotherapy (**Figure 5D**, p=1.39e-03) and endocrinotherapy (**Figure 5E**, p=2.853e-04) as well as radiotherapy (**Figure 5F**, p=1.303e-07). From this aspect, the BMscore signature can be used effectively to identify therapy-resistant breast cancer patients.

### Identification and evaluation of the independent prognostic value of the BMscore in breast cancer

**Figure 6:**
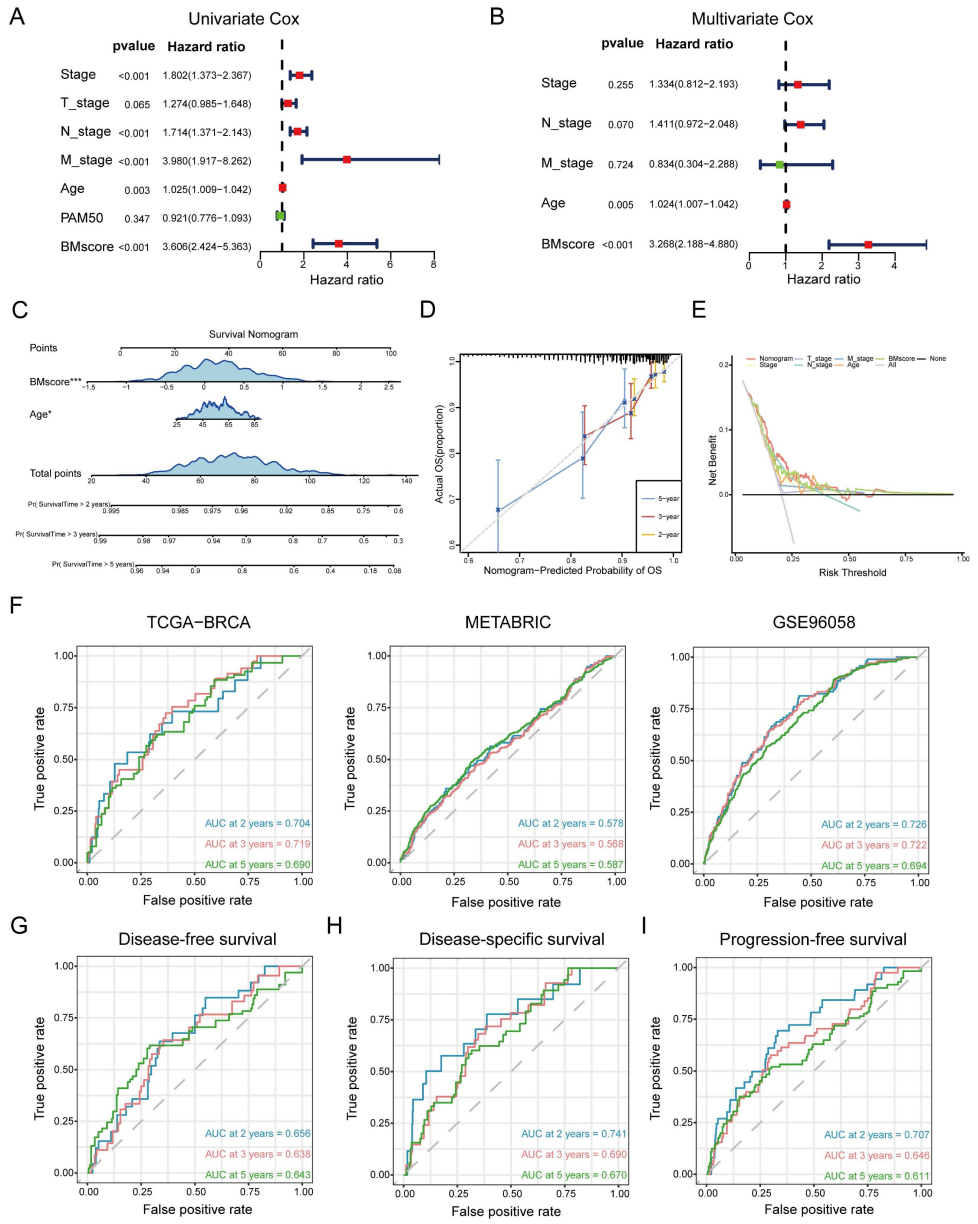
Generation and validation of a prognostic nomogram based on the BMscore. (A and B) Univariate (A) and multivariate (B) Cox regression analyses in breast cancer patients from TCGA-BRCA. (C) Development of a nomogram to predict OS probabilities. (D, E and F) The calibration curve (D), DCA curve (E) and ROC curves (F) of the model to evaluate prediction accuracy for OS of breast cancer patients. (G, H and I) The ROC curves of the model to predict DFS (G), DSS (H) and PFS (I) in patients of TCGA-BRCA.

In summary, the BMscore signature performed well in forecasting prognosis as well as therapy response among breast cancer patients. However, additional research remains necessary to investigate whether the BMscore could serve as an independent and adverse prognostic predictor affecting survival in breast cancer. In addition to the BMscore, multiple risk factors (age, T stage, N stage, M stage and PAM50 subtype) were enrolled together to carry out univariate and multivariate Cox analyses for OS in TCGA-BRCA. The univariate analysis identified BMscore (p<0.001), stage (p<0.001), age (p=0.003), N stage (p<0.001) and M stage (p<0.001) as independent risk factors for OS **(Figure 6A)**, and subsequently, age (p=0.005) and BMscore (p<0.001) remained as independent predictors in multivariate analysis for OS **(Figure 6B)**. Then a prognostic nomogram incorporating these two factors was constructed to predict unfavorable OS **(Figure 6C)**. The calibration **(Figure 6D)** and DCA curves** (Figure 6E)** for the nomogram in TCGA-BRCA were drawn to assess the prediction performance and clinical utility, which indicated that the nomogram was able to provide valuable judgment for prognosis.

Consistently, ROC curves were applied to represent the discrimination ability of the nomogram. In TCGA-BRCA, the AUC values of 2-, 3- and 5-year OS were 0.704, 0.719 and 0.690, respectively. In METABRIC, the AUC values of 2-, 3- and 5-year OS were 0.578, 0.568 and 0.587, respectively. In GSE96058, the AUC values of 2-, 3- and 5-year OS were 0.726, 0.722 and 0.694, respectively. These results convincingly proved that the nomogram showed predictive power in predicting OS across all three cohorts (AUC > 0.5) **(Figure 6F)**. Indeed, we also observed that the nomogram had good sensitivity and specificity in predicting other survival outcomes in TCGA-BRCA, including DFS (**Figure 6G**, 2-, 3- and 5-year AUCs: 0.656, 0.638, and 0.643), DSS (**Figure 6H**, 2-, 3- and 5-year AUCs: 0.741, 0.690, and 0.670) and PFS (**Figure 6I**, 2-, 3- and 5-year AUCs: 0.707, 0.646, and 0.611). At this point, we concluded that the BMscore could function as an independent prognostic indicator for breast cancer.

### Nonnegligible impact of the BMscore on the breast cancer immune microenvironment

**Figure 7:**
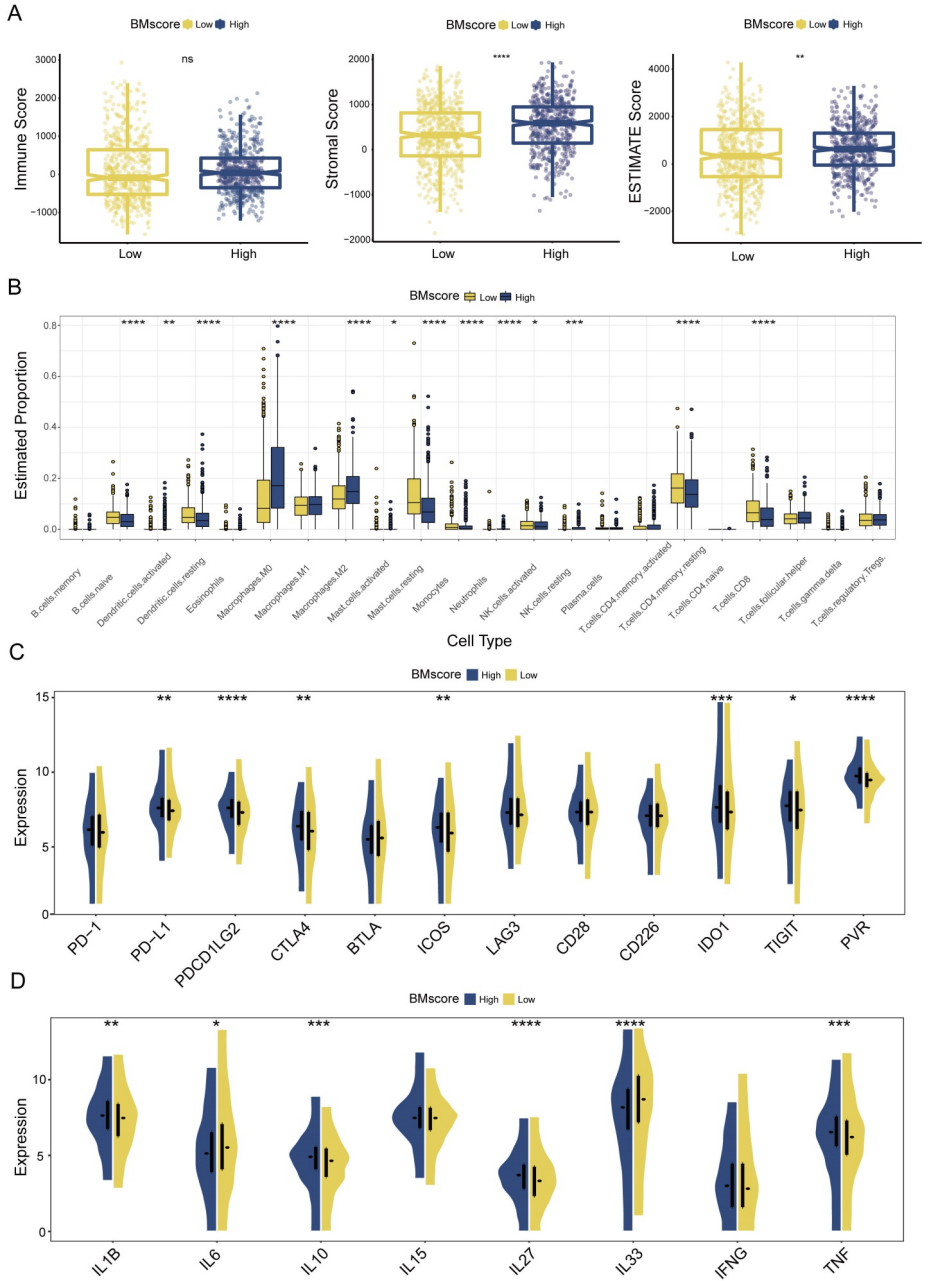
Estimation of tumor immune infiltration of breast cancer according to the BMscore. (A) Scatterplots of the immune score, stromal score and ESTIMATE score between the high and low BMscore groups. (B) Infiltrating degree of 22 immune cells between the high and low BMscore groups. (C) Expression changes of immune checkpoint markers within different BMscore levels. (D) Changes in cytokine mRNA expression levels based on BMscore levels.

Given the major role of tumor immune microenvironment in breast cancer, we next assessed the effect of the BMscore on tumor immune infiltration. According to the ESTIMATE algorithm, higher stromal score and ESTIMATE score suggested a greater degree of stromal cell infiltration in high-BMscore tumors **(Figure 7A)**. Of note, the immune score did not differ significantly across different BMscore subgroups. This finding revealed that breast tumor with high BMscore had a low purity, which might facilitate poorer outcomes in this subpopulation.

Although the BM and immune cells are both essential parts of the tumor microenvironment, their connection is still ill-defined, which prompted the subsequent part of our analyses. Using the CIBERSORT algorithm, we inferred the infiltration levels of 22 immune cells within breast cancer samples of different BMscore levels in all three cohorts. In the high BMscore group of TCGA-BRCA, a particularly impressive increase was observed in infiltrating M0 macrophages, M2 macrophages, and resting NK cells, while a significant reduction was observed in the infiltration of naive B cells, resting dendritic cells, resting mast cells, monocytes, activated NK cells, resting CD4^+^ memory T cells and CD8^+^ T cells **(Figure 7B)**. Combining the results of METABRIC **(Figure S3A)** and GSE96058 **(Figure S4A)**, it could be summarized that increasing infiltrations of M0 macrophages, M2 macrophages, and regulatory T cells were presented in breast cancer with high BMscores, which further confirmed the oncogenic and immunosuppressive role of BM.

Meanwhile, we analyzed the correlation between BMscore levels and expressions of immune checkpoints and cytokines. The results showed that high BMscores signified upregulated expressions of immune checkpoint molecules, including CTLA4, IDO1, ICOS and PVR, which drew collectively from TCGA-BRCA **(Figure 7C)**, METABRIC **(Figure S3B)** and GSE96058 **(Figure S4B)**. The above clue indicated that patients with high BMscore may be more sensitive to immune checkpoint blockade therapy, especially anti-CTLA4 treatment. In terms of cytokines, we noticed that TNF, IL27 and IL1B highly expressed in the high BMscore subgroup while IL33 and IL6 reduced obviously after incorporating data from all cohorts (TCGA-BRCA, **Figure 7D**; METABRIC, **Figure S3C**; GSE96058, **Figure S4C**). Taken together, the results reveal that the expression of tumor-promoting cytokines contributed to the prognostic features of breast cancer patients who had high BMscores.

**Figure 8:**
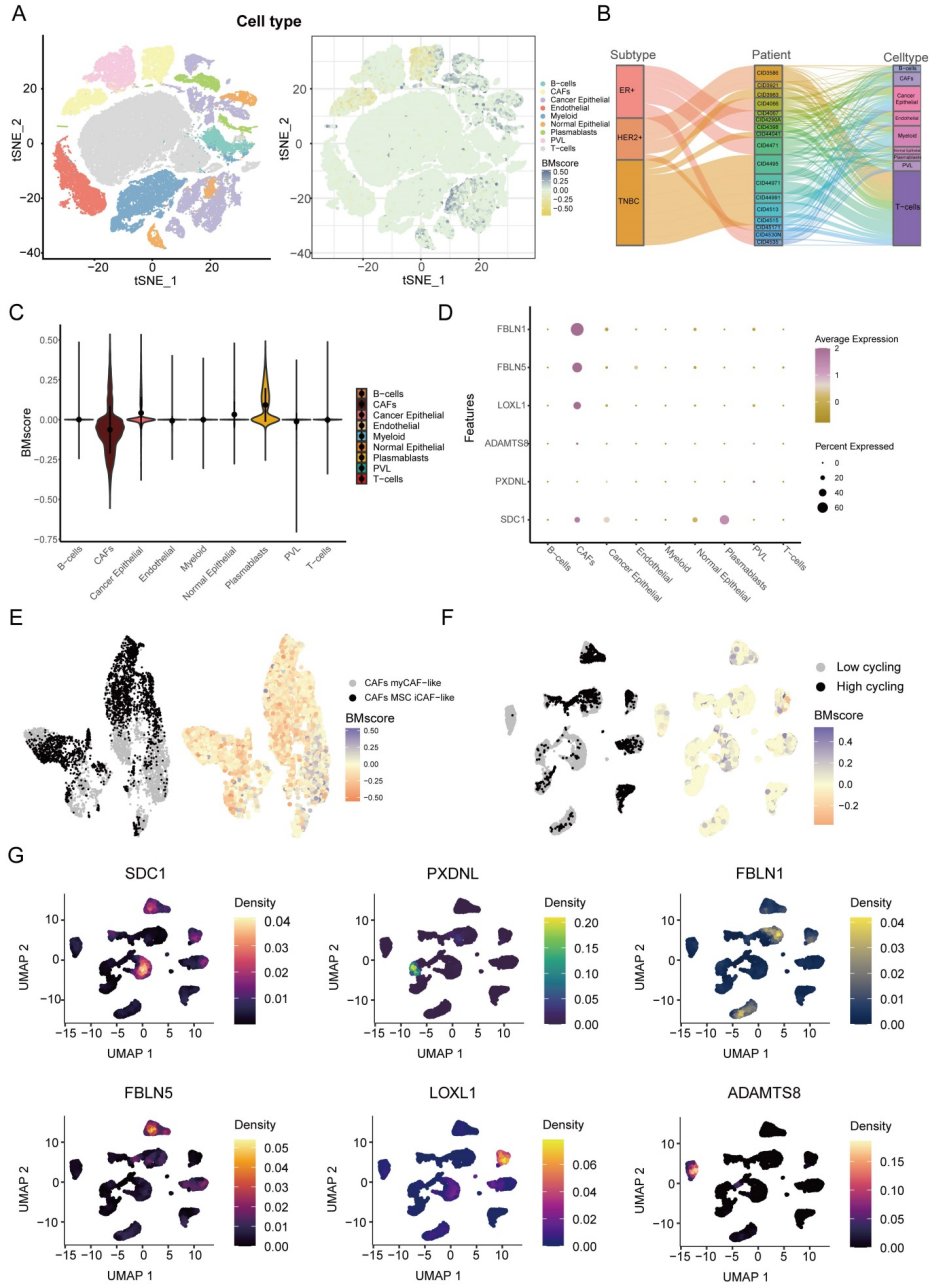
Dissection of tumor microenvironment based on the BMscore signature. (A) t-SNE plot visualization of all cell subtypes and the distribution of the BMscore from 26 breast cancer patients. Different cell subtypes were annotated by Seurat algorithm. (B) Sankey diagram shows the interrelationship between breast cancer subtypes, patients ID, and cell types in breast cancer patients. (C) Violin plot of the CDI value in different cell types. (D) Bubble plot of the average and percent expression of model genes in different cell subtypes. (E) UMAP plot visualization of the distribution of the CAF subpopulation and BMscore. (F) UMAP plot visualization of high and low cell cycling cancer epithelial cells and expression of model genes.

Next, we explored the detailed distribution of the BMscore in breast cancer using single-cell RNA transcriptome data. We annotated the major cell types in GSE176078, then we found that the BMscore in cancer epithelial cells, CAFs and plasmablasts was significantly different from that in other cell types **(Figure 8A)**. Since there are multiple subtypes of breast cancer, we filtered out patients with cell counts less than 2000 and showed the proportion of patients in the three breast cancer subtypes and the proportion of each cell type for each patient using a Sankey diagram **(Figure 8B)**. The violin plot illustrated the differences more clearly in BMscores across cell types, consistently showing that the BMscore was higher in tumor cells **(Figure 8C)**. Then, the dot plot provided a visualization of the expression of six model genes in each cell type and the result was consistent with the previous description **(Figure 8D)**. Since the BMscore varied significantly within CAF cells, we further annotated CAF cells for classification, and we found that most cells with a high BMscore were classified into myCAF-like cells, which were most concentrated in the invasive fraction of breast tumors **(Figure 8E)** 36. In addition, to investigate the relationship between the internal heterogeneity of cancer epithelial cells and BMscore and model genes, we divided cancer epithelial cells into high and low cell cycling groups and displayed the distribution of the BMscore and expression of model genes in these groups **(Figure 8F, G)**.

### Positive correlation between BMscore and EMT activity in breast cancer

**Figure 9:**
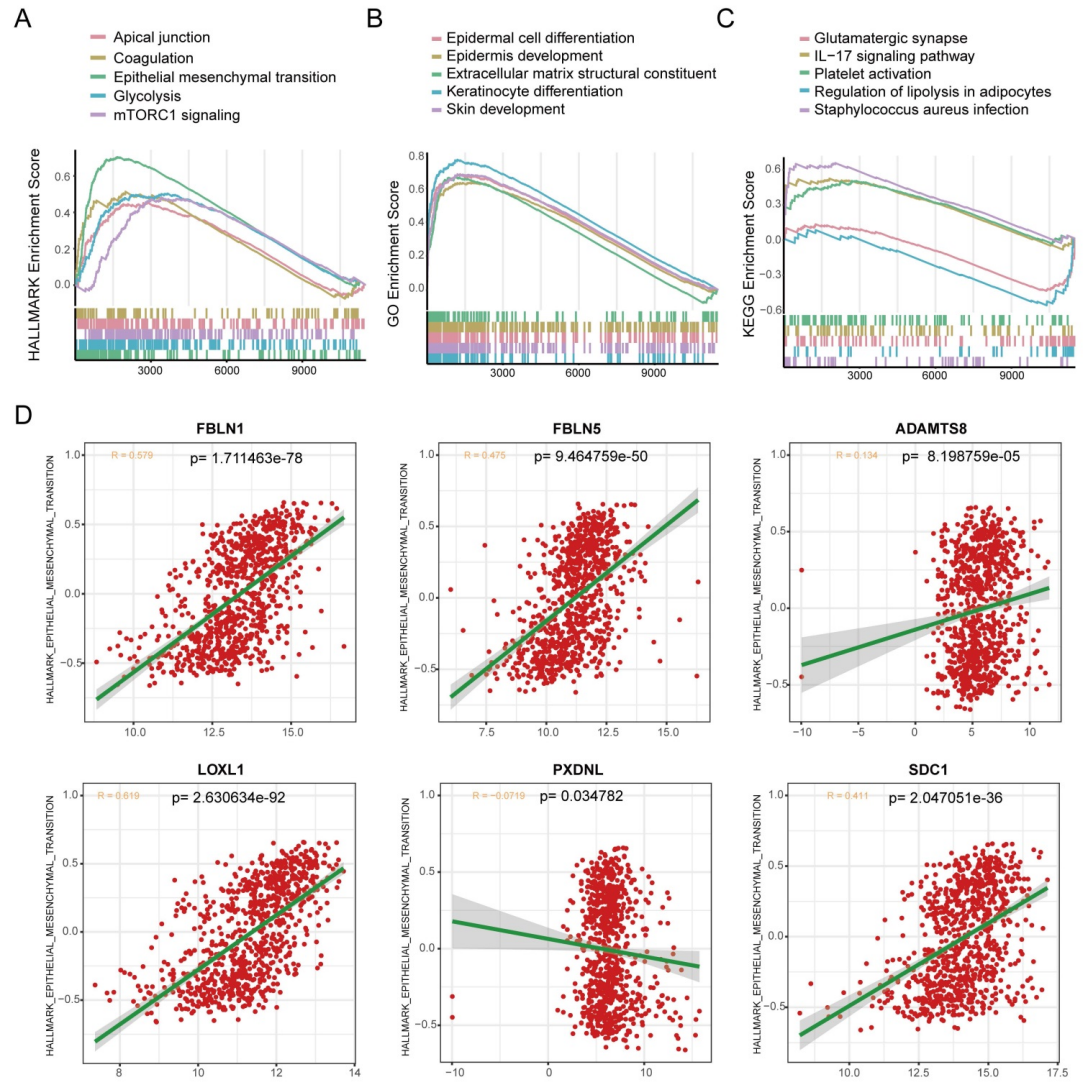
Exploration of the correlation between the BMscore and the EMT pathway. (A, B and C) Gene set enrichment analyses (GSEA) of the hallmark gene set (A), GO enrichment (B) and KEGG pathway enrichment (C). (D) Scatterplots of expression correlation between the six signature genes and the EMT pathway in the hallmark gene set.

BM loss has been regarded as an essential step leading to tumor malignancy 37, which prompted us to examine the correlation between the BMscore and EMT. For functional enrichment analysis, the hallmark pathways enriched in the high BMscore group included apical junction, coagulation, epithelial-mesenchymal transition, glycolysis and mTORC1 signaling **(Figure 9A)**. In addition, GO terms of high BMscore group contained epidermal cell differentiation, epidermis development, extracellular matrix structural constituent, keratinocyte differentiation and skin development **(Figure 9B)**. Also, KEGG enrichment analysis showed that a high BMscore was related to glutamatergic synapses, the IL-17 signaling pathway, platelet activation, regulation of lipolysis in adipocytes and Staphylococcus aureus infection **(Figure 9C)**. Notably, the results provided clues for the significance of BM in breast cancer progression and metastasis.

Since BM deficiency makes patients prone to tumor cell metastasis, and EMT plays a prominent role in breast cancer metastasis 38, we wondered if there was any association between the BMscore and EMT activity. By scatter plots of correlation analyses **(Figure 9D)**, we found that there was a strongly positive correlation between the EMT pathway and the expression of FBLN1 (R = 0.579, p=1.711463e-78), FBLN5 (R = 0.475, p=9.464759e-50), LOXL1 (R = 0.619, p= 2.630634e-92) and SDC1 (R = 0.411, p=2.047051e-36).

**Figure 10:**
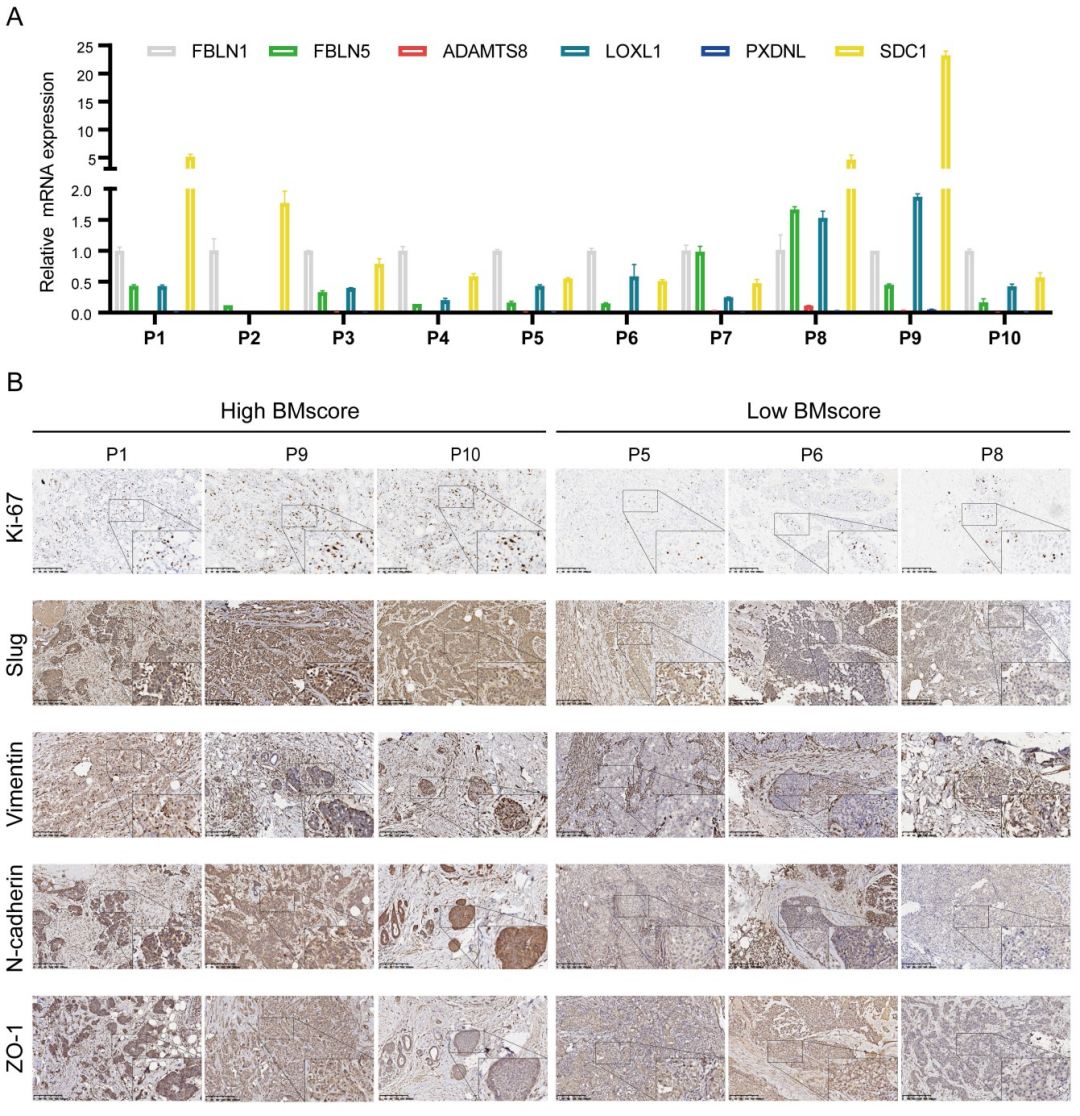
Verification of the difference between breast cancer tissues with high and low BMscores. (A) The mRNA levels of the six signature genes were determined in 10 paired breast cancer tissues and adjacent normal samples. (B) Immunohistochemical comparison of Ki-67 and EMT-related markers between tissues with high and low BMscores.

Combined with our earlier finding that the EMT signaling pathway was enriched in high BMscore group, we hypothesized that the BMscore was positively correlated with EMT levels. To validate our conjecture, we first detected the mRNA levels of 6 BMscore-contained genes in 10 human breast cancer samples **(Figure 10A)**. Afterward, the BMscore was calculated for each sample using the previously described formula. We selected 6 samples with the three top-scored and the top three lowest of BMscore, and frozen sections of these tumors were tested by immunohistochemistry to detect the protein levels of Ki-67 and several EMT markers, including slug, vimentin, N-cadherin and ZO-1 **(Figure 10B)**. Unexpectedly, we discovered that these proteins were relatively more abundant in tissues with high BMscores, which uncovered a strong positive relationship between the BMscore and EMT activity. Along these lines, breast cancer patients with a high BMscore might be at greater risk of EMT-mediated metastasis.

### SDC1 serves as a key player in the BMscore as well as a tumor promoter in breast cancer

**Figure 11:**
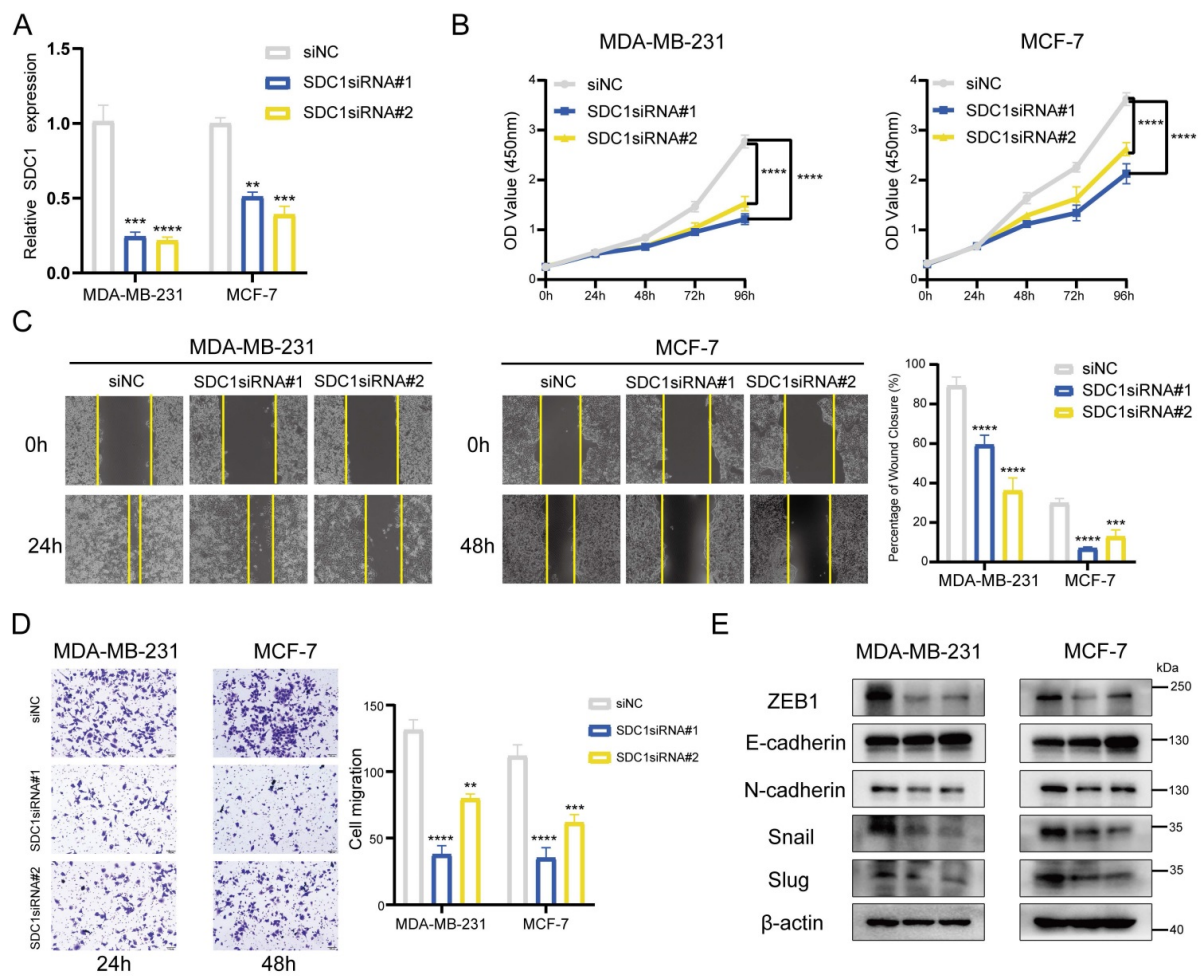
Effects of SDC1 silencing on the growth and migration of breast cancer cells. (A) Detection of SDC1 mRNA knockdown by RT-qPCR. (B) Growth curves of MDA-MB-231 and MCF-7 cells with SDC1 knockdown as evaluated by the CCK8 assay. (C and D) The scratch (C) and transwell (D) assays demonstrating the effects of SDC1 inhibition on cell migration. (E) The detection of EMT-associated proteins after SDC1 knockdown.

By following the aggregate of all the above findings, we determined SDC1 as the key gene associated with the BMscore signature and performed additional experiments *in vitro*. With siRNA-mediated knockdown, SDC1 mRNA levels were reduced in MDA-MB-231 and MCF-7 cells **(Figure 11A)**. The CCK8 cell proliferation assays showed a significant growth inhibition in both MDA-MB-231 and MCF-7 cell lines with SDC1 reduction** (Figure 11B)**. Similarly, decreased SDC1 induced migratory inhibition in breast cancer cells, as demonstrated by scratch wound healing **(Figure 11C)** and transwell migration assays **(Figure 11D)**. Moreover, the protein levels of EMT markers were detected in siRNA-transfected cells to verify whether SDC1 alteration resulted in changes in EMT marker genes. As anticipated, SDC1 silencing suppressed the protein expression of snail, slug, N-cadherin and ZEB1, while a corresponding increase of E-cadherin was observed **(Figure 11E)**. Up to this point, our data confirmed the key role of SDC1 in the EMT-related progression of breast cancer.

## Discussion

There has been a revival of interest in the role of BM in tumor progression and metastasis in recent years. Thus, we decided to explore the clinical value of BM-related genes in breast cancer. In this study, BM genes with differential expression and prognostic correlation in breast cancer were selected as promising prognostic biomarkers for breast cancer, including FBLN1, FBLN5, ADAMTS8, LOXL1, SDC1 and PXDNL.

The fibulin protein family is prevalent in the ECM and performs a pivotal role in the composition and stabilization of the basement membrane 39. Among these candidate BM genes, FBLN5 (fibulin-5) has been reported to be a tumor suppressor in tumorigenesis 40, such as in lung cancer 41, breast cancer 42, and hepatocellular carcinoma 43. FBLN1 (fibulin-1) was determined to be a prognostic factor in patients with gastric cancer by inhibiting cell growth and promoting apoptosis 44. For ADAMTS8, it appears that some cancers have decreased ADAMTS8 expression 45, 46. Meanwhile, ADAMTS8 exhibited an inhibitory influence on tumor proliferation and invasion 47-49. The above arguments were well demonstrated in breast cancer in our study. LOXL1, a member of LOX-like (LOXL) proteins, was found to be an aggressive player in glioma 50 and non-small cell lung cancer 51. Furthermore, the suppressive function of LOXL1 was also revealed in bladder cancer and colorectal cancer 52, 53. In our analysis, further clarity is needed to verify whether LOXL1 is a friend or foe in breast cancer. For the remaining two BM genes (PXDNL and SDC1), our data indicated that high expression was associated with unfavorable prognoses in breast cancer. As a surface protein on endothelial cells, SDC1 (syndecan-1) is the best characterized molecule of the syndecan family. Dysregulated expression of SDC1 in cancer has prognostic relevance and clinical significance 54. In breast cancer, high SDC1 expression has been shown to be associated with aggressive tumors and poor prognosis 55. Other studies have suggested that SDC1 functions as a strongly useful biomarker for the prognosis of aggressive breast cancer 56, 57. This conclusion fully agrees with our observations, and a deeper understanding of the molecular mechanism of SDC1 in breast cancer should be provided in the future. Alternatively, in addition to the important clue unraveled in our study, the relationship between PXDNL and cancer has remained unknown until now, and more investigations are warranted.

Based on the six candidate BM genes, we established the BMscore and investigated its prognostic and predictive value, as well as its strong associations with breast cancer. From the results, there is a clear separation in the survival curves between patients with high- and low- BMscores, and our study suggested that patients with high BMscores have worse clinical status and survival outcomes than those with low BMscores. In addition, failure of adjuvant therapy was more likely to be observed in breast cancer patients with high BMscores. We hope that a larger prospective study will support this conclusion in the future. Subsequently, we constructed a prognostic nomogram through integrating the BMscore and age, which showed good performance in the prediction and accuracy of survival probabilities.

Since ECM is a critical component of the tumor microenvironment, in which tumor cells interact with infiltrating immune cells. Thus, we assessed the changes in tumor immune infiltration characteristics according to different BMscore levels in breast cancer. Briefly, breast tumors with high BMscores have lower tumor purity, increased infiltration of macrophages and upregulated expression of several immune checkpoint molecules, which implies that these patients may have a poor response to immune therapy. Notably, single-cell data analysis also hinted that CAFs were concentrated in breast tumors with low BMscores. In fact, our study sheds new light on the immune effects of BM genes, which still require further experimentation to gain definitive proof.

Despite the advantages of the current study, there are some limitations as well. According to the reviewed analyses, our conclusions provided clues related to the BM and breast cancer, which should be supported by direct experimental evidence in future. Moreover, our results showed the striking changes in the molecular characteristics of breast cancer, and more specific investigation are required to verify these observed relationships. In any case, we will give more attention to emerging additional data so as to further verify our proposed signature in the future.

## Conclusions

In conclusion, the BM-related gene signature presented in this study is a practical prognostic indicator that can enable a noticeable difference in the appraisal of the survival outcomes for breast cancer patients. Additionally, clinical tissue samples from breast cancer patients verified the strongly positive correlation between the BMscore and the EMT activity, which strengthened the novel implications of basement membrane in breast cancer.

## Supplementary Material

Supplementary figures.

## Abbreviations

BM: basement membrane
EMT: epithelial mesenchymal transition
ECM: extracellular matrix
GSEA: gene set enrichment analysis
OS: overall survival
DFS: disease-free survival
DSS: disease-specific survival
PFS: progression-free survival
