# Supplementary material for: Novel Implication of the Basement Membrane for Breast Cancer Outcome and Immune Infiltration
Source: Int J Biol Sci. 2023 Mar 5;19(5):1645–63. doi: 10.7150/ijbs.81939 (PMC10086744; doi:10.7150/ijbs.81939)
Supplement: Supplementary file 1 — Supplementary figures. [file ijbsv19p1645s1.pdf]

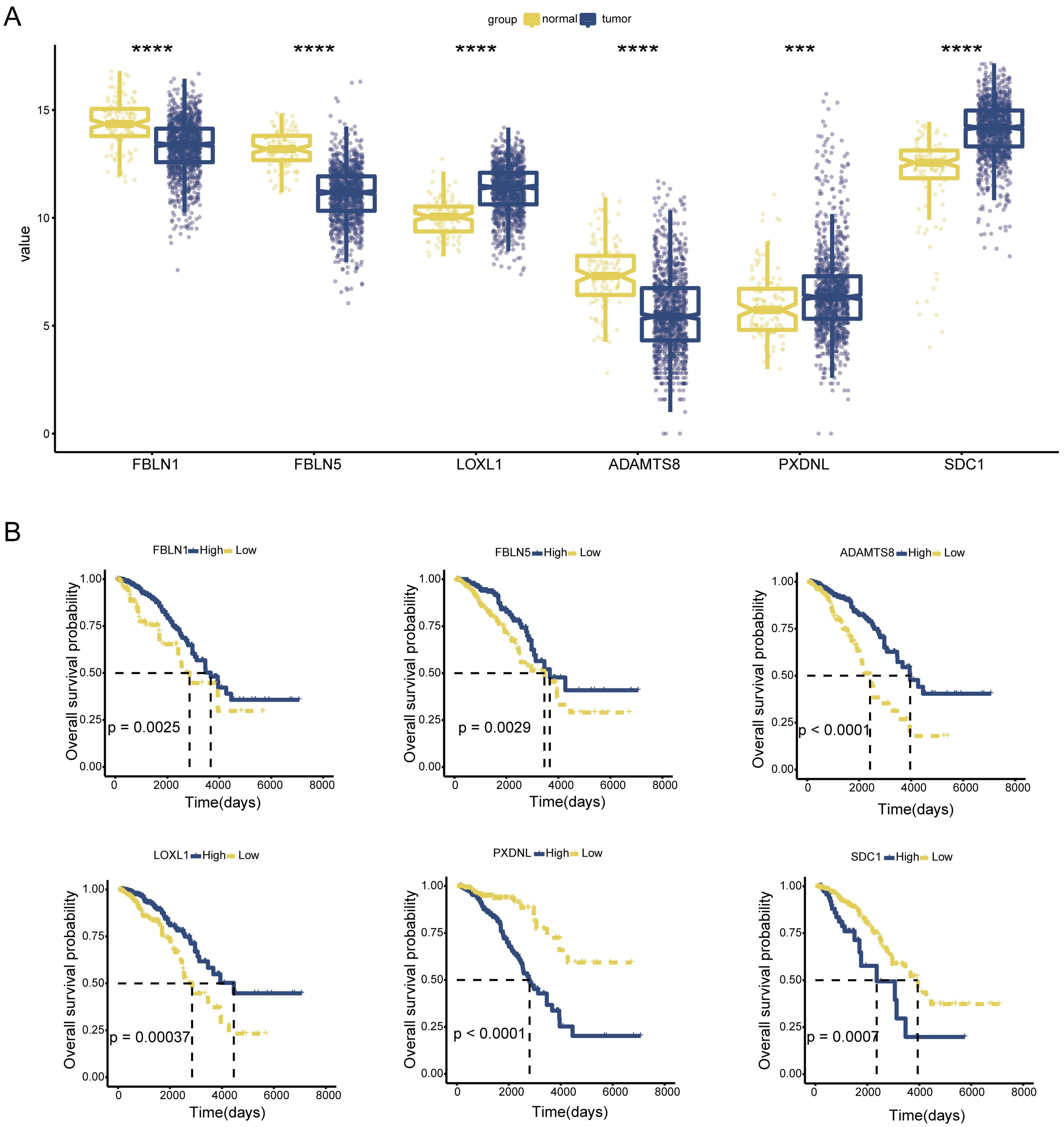

A
group normal tumor
****
****
****
****
***
****
value
15
10
5
0
FBLN1
FBLN5
LOXL1
ADAMTS8
PXDNL
SDC1
B
FBLN1 High Low
p = 0.0025
FBLN5 High Low
p = 0.0029
ADAMTS8 High Low
p < 0.0001
LOXL1 High Low
p = 0.00037
PXDNL High Low
p < 0.0001
SDC1 High Low
p = 0.0007
Overall survival probability
Time(days)

Figure S1

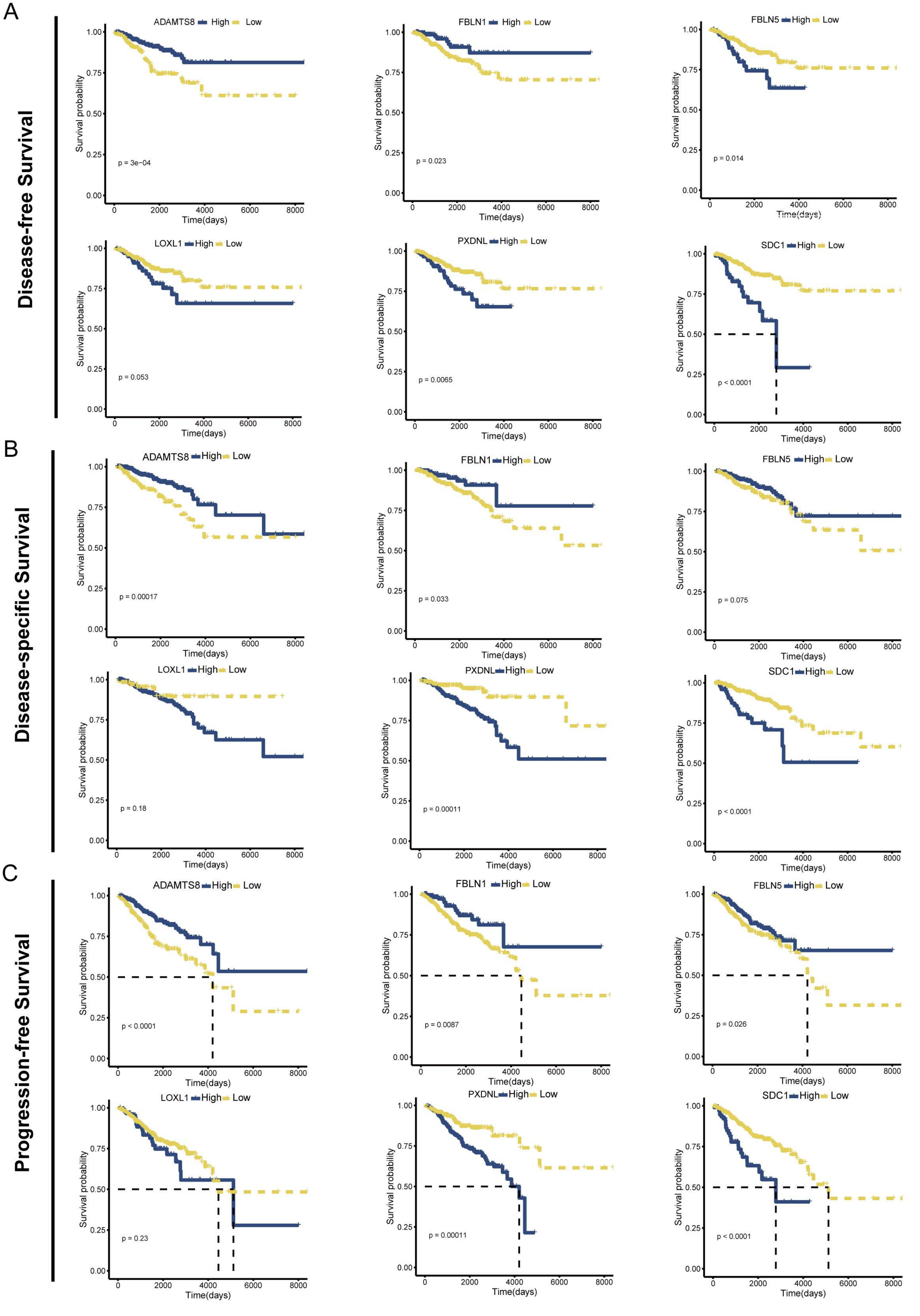
A
Disease-free Survival
ADAMTS8 High Low
p = 3e−04
FBLN1 High Low
p = 0.023
FBLN5 High Low
p = 0.014
LOXL1 High Low
p = 0.053
PXDNL High Low
p = 0.0065
SDC1 High Low
p < 0.0001
B
Disease-specific Survival
ADAMTS8 High Low
p = 0.00017
FBLN1 High Low
p = 0.033
FBLN5 High Low
p = 0.075
LOXL1 High Low
p = 0.18
PXDNL High Low
p = 0.00011
SDC1 High Low
p < 0.0001
C
Progression-free Survival
ADAMTS8 High Low
p < 0.0001
FBLN1 High Low
p = 0.0087
FBLN5 High Low
p = 0.026
LOXL1 High Low
p = 0.23
PXDNL High Low
p = 0.00011
SDC1 High Low
p < 0.0001
Survival probability
Time(days)
0.00
0.25
0.50
0.75
1.00
0
2000
4000
6000
8000

Figure S2

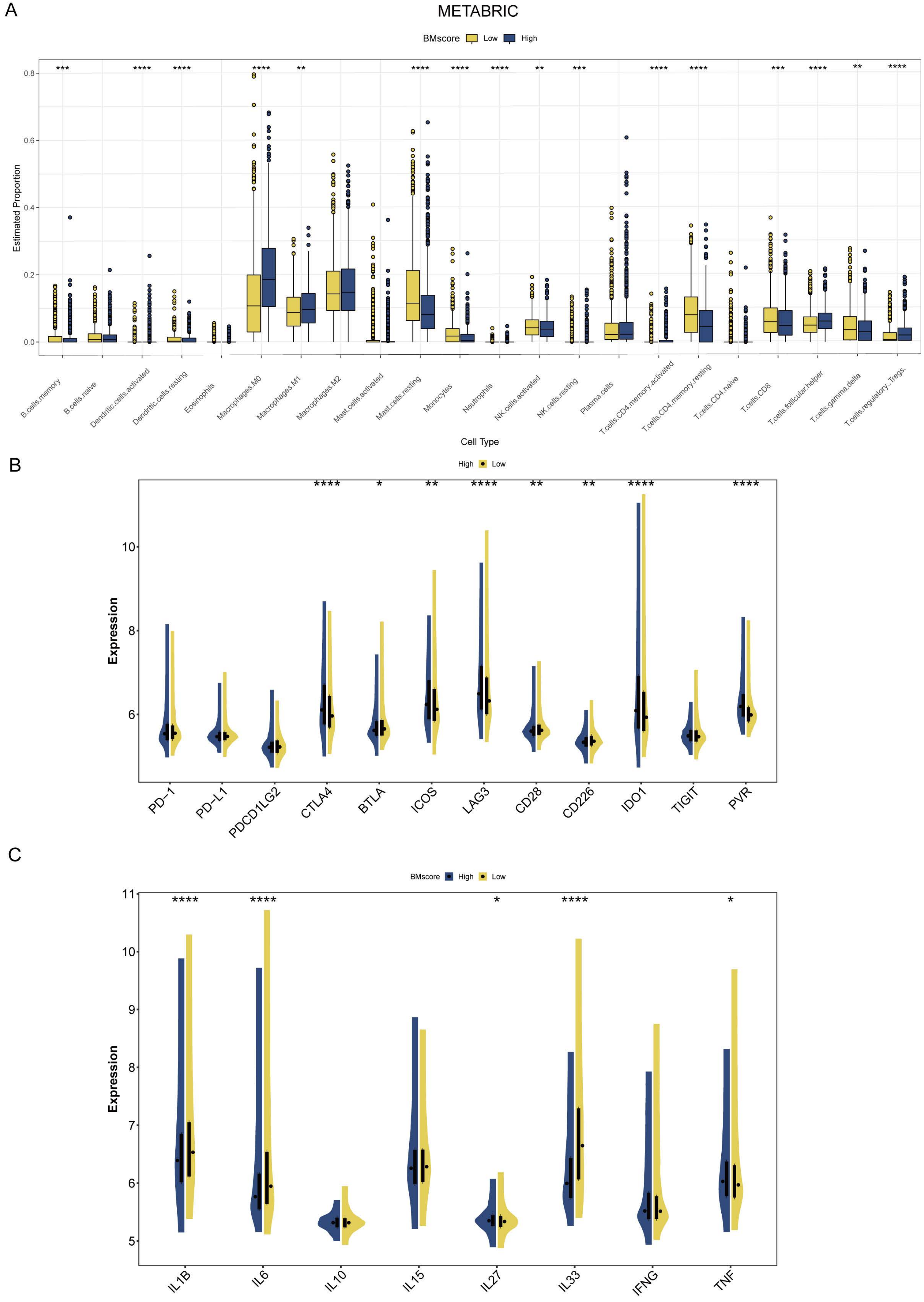

A
METABRIC
BMscore Low High
Estimated Proportion
0.8
0.6
0.4
0.2
0.0
*** **** **** **** ** **** **** **** ** *** **** **** *** **** ** ****
B.cells.memory
B.cells.naive
Dendritic.cells.activated
Dendritic.cells.resting
Eosinophils
Macrophages.M0
Macrophages.M1
Macrophages.M2
Mast.cells.activated
Mast.cells.resting
Monocytes
Neutrophils
NK.cells.activated
NK.cells.resting
Plasma.cells
T.cells.CD4.memory.activated
T.cells.CD4.memory.resting
T.cells.CD4.naive
T.cells.CD8
T.cells.follicular.helper
T.cells.gamma.delta
T.cells.regulatory..Tregs.
Cell Type
B
High Low
Expression
10
8
6
**** * ** **** ** ** **** ****
PD−1
PD−L1
PDCD1LG2
CTLA4
BTLA
ICOS
LAG3
CD28
CD226
IDO1
TIGIT
PVR
C
BMscore High Low
Expression
11
10
9
8
7
6
5
**** **** * **** *
IL1B
IL6
IL10
IL15
IL27
IL33
IFNG
TNF

Figure S3

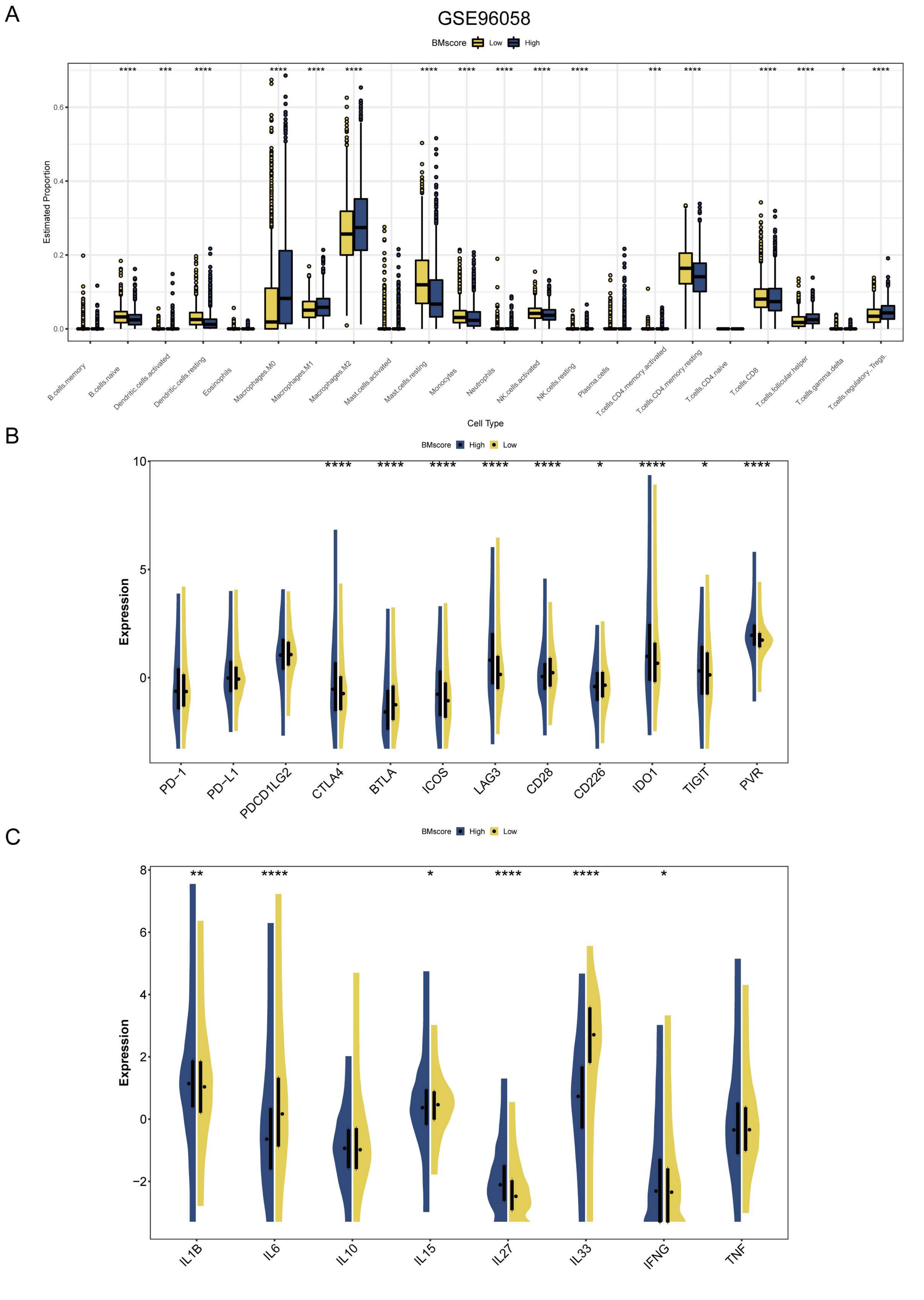
A
GSE96058
BMscore Low High
Estimated Proportion
0.0
0.2
0.4
0.6
**** *** **** **** **** **** **** **** **** **** **** *** **** **** **** * ****
B.cells.memory
B.cells.naive
Dendritic.cells.activated
Dendritic.cells.resting
Eosinophils
Macrophages.M0
Macrophages.M1
Macrophages.M2
Mast.cells.activated
Mast.cells.resting
Monocytes
Neutrophils
NK.cells.activated
NK.cells.resting
Plasma.cells
T.cells.CD4.memory.activated
T.cells.CD4.memory.resting
T.cells.CD4.naive
T.cells.CD8
T.cells.follicular.helper
T.cells.gamma.delta
T.cells.regulatory..Tregs.
Cell Type
B
BMscore High Low
Expression
10
5
0
**** **** **** **** **** * **** * ****
PD−1
PD−L1
PDCD1LG2
CTLA4
BTLA
ICOS
LAG3
CD28
CD226
IDO1
TIGIT
PVR
C
BMscore High Low
Expression
8
6
4
2
0
−2
** **** * **** **** *
IL1B
IL6
IL10
IL15
IL27
IL33
IFNG
TNF

Figure S4

Supplementary figure legends:

Figure S1. Description of the prognosis-related BM genes in breast cancer. (A) Estimation over the expression levels of six signature-contained BM genes in TCGA cohort. (B) The KM survival curves of six signature-contained BM genes based on expression levels and OS.

Figure S2. The KM survival curves of six signature-contained BM genes based on the mRNA expression levels for disease-free survival (A), disease-specific survival (B) and progression-free survival (C).

Figure S3. Tumor microenvironment assessment in METABRIC cohort. (A) Infiltrating degree of 22 immune cells between high and low BMscore groups. (B) Expression changes of immune checkpoint markers within different BMscore levels. (C) Changes of the cytokine mRNA expression levels based on BMscore levels.

Figure S4. The TME landscapes between high- and low-BMscore groups were estimated in GSE96058 cohort. (A) Infiltrating degree of 22 immune cells between high and low BMscore groups. (B) Expression changes of immune checkpoint markers within different BMscore levels. (C) Changes of the cytokine mRNA expression levels based on BMscore levels.
